# Multivariable Model to Predict an ACTH Stimulation Test to Diagnose Adrenal Insufficiency Using Previous Test Results

**DOI:** 10.1210/jendso/bvad127

**Published:** 2023-10-07

**Authors:** Neil Richard Lawrence, Muhammad Fahad Arshad, Riccardo Pofi, Sean Ashby, Jeremy Dawson, Jeremy W Tomlinson, John Newell-Price, Richard J Ross, Charlotte J Elder, Miguel Debono

**Affiliations:** Division of Clinical Medicine, School of Medicine and Population Health, University of Sheffield, Sheffield S10 2TN, UK; Paediatric Endocrinology Department, Sheffield Children's NHS Foundation Trust, Sheffield S10 2TH, UK; Division of Clinical Medicine, School of Medicine and Population Health, University of Sheffield, Sheffield S10 2TN, UK; Endocrinology Department, Sheffield Teaching Hospitals NHS Foundation Trust, Sheffield S10 2JF, UK; Oxford Centre for Diabetes, Endocrinology and Metabolism, Churchill Hospital, University Hospitals NHS Trust, Oxford OX3 9DU, UK; Division of Clinical Medicine, School of Medicine and Population Health, University of Sheffield, Sheffield S10 2TN, UK; Division of Clinical Medicine, School of Medicine and Population Health, University of Sheffield, Sheffield S10 2TN, UK; Oxford Centre for Diabetes, Endocrinology and Metabolism, Churchill Hospital, University Hospitals NHS Trust, Oxford OX3 9DU, UK; Oxford Centre for Diabetes, Endocrinology and Metabolism, NIHR Oxford Biomedical Research Centre, University of Oxford, Oxford OX3 9DU, UK; Division of Clinical Medicine, School of Medicine and Population Health, University of Sheffield, Sheffield S10 2TN, UK; Endocrinology Department, Sheffield Teaching Hospitals NHS Foundation Trust, Sheffield S10 2JF, UK; Division of Clinical Medicine, School of Medicine and Population Health, University of Sheffield, Sheffield S10 2TN, UK; Division of Clinical Medicine, School of Medicine and Population Health, University of Sheffield, Sheffield S10 2TN, UK; Paediatric Endocrinology Department, Sheffield Children's NHS Foundation Trust, Sheffield S10 2TH, UK; Division of Clinical Medicine, School of Medicine and Population Health, University of Sheffield, Sheffield S10 2TN, UK; Endocrinology Department, Sheffield Teaching Hospitals NHS Foundation Trust, Sheffield S10 2JF, UK

**Keywords:** adrenal insufficiency, short Synacthen test, adrenocorticotropin stimulation test, predictive model, cortisol

## Abstract

**Context:**

The adrenocorticotropin hormone stimulation test (AST) is used to diagnose adrenal insufficiency, and is often repeated in patients when monitoring recovery of the hypothalamo–pituitary–adrenal axis.

**Objective:**

To develop and validate a prediction model that uses previous AST results with new baseline cortisol to predict the result of a new AST.

**Methods:**

This was a retrospective, longitudinal cohort study in patients who had undergone at least 2 ASTs, using polynomial regression with backwards variable selection, at a Tertiary UK adult endocrinology center. Model was developed from 258 paired ASTs over 5 years in 175 adults (mean age 52.4 years, SD 16.4), then validated on data from 111 patients over 1 year (51.8, 17.5) from the same center, data collected after model development. Candidate prediction variables included previous test baseline adrenocorticotropin hormone (ACTH), previous test baseline and 30-minute cortisol, days between tests, and new baseline ACTH and cortisol used with calculated cortisol/ACTH ratios to assess 8 candidate predictors. The main outcome measure was a new test cortisol measured 30 minutes after Synacthen administration.

**Results:**

Using 258 sequential ASTs from 175 patients for model development and 111 patient tests for model validation, previous baseline cortisol, previous 30-minute cortisol and new baseline cortisol were superior at predicting new 30-minute cortisol (*R*^2^ = 0.71 [0.49-0.93], area under the curve [AUC] = 0.97 [0.94-1.0]) than new baseline cortisol alone (*R*^2^ = 0.53 [0.22-0.84], AUC = 0.88 [0.81-0.95]).

**Conclusion:**

Results of a previous AST can be objectively combined with new early-morning cortisol to predict the results of a new AST better than new early-morning cortisol alone. An online calculator is available at https://endocrinology.shinyapps.io/sheffield_sst_calculator/ for external validation.

Adrenal insufficiency (AI) is a failure of the adrenal glands to produce sufficient cortisol, with presentations ranging from nonspecific symptoms to life-threatening adrenal crisis. The 250-µg short Synacthen test or adrenocorticotropin stimulation test (AST) is the most widely used diagnostic test for AI worldwide, with pass or fail dictated by an assay-dependent threshold level of cortisol after administration of synthetic adrenocorticotropin hormone (ACTH) [1-3].

AI is classified into 3 different types. A raised baseline ACTH in the context of a failed AST indicates an adrenal cause (primary AI). Normal or low ACTH suggests impaired pituitary function, caused either by pituitary disease impairing secretion (secondary AI) or by reduced corticotropin-releasing hormone from the hypothalamus (tertiary AI), the latter caused most frequently by chronic exogenous glucocorticoids or opioid exposure [4, 5]. The use of systemic glucocorticoids is increasing in both Europe and the United States, with a prevalence of between 1% to 3% and up to 10% in elderly people [6, 7]. With up to 50% of patients on systemic glucocorticoids at risk of AI, the prevalence of AI in the general population is likely to be high [5]. Patients weaning from systemic glucocorticoids have a high risk of adrenal suppression related to cumulative doses and time on glucocorticoids [8]. Whether a patient will recover adrenal function and how long this will take is difficult to predict [9]. To avoid the long-term negative impact of protracted periods on glucocorticoids, sequential morning cortisol and ASTs are often performed to guide the weaning process [10, 11]. This increases the burden of resource- and time-consuming dynamic testing on patients and health care providers.

Applying thresholds to measurements of unstimulated early-morning cortisol has been proposed to reduce the number of ASTs performed. Values of less than 20 nmol/L to 200 nmol/L are cited as predictive for AI and levels of greater than 330 nmol/L to 506 nmol/L have been proposed to assume adequate adrenal function [12-18]. These cutoffs depend on the desired sensitivity or specificity, with levels in between considered equivocal. Thresholds have also been proposed using data from sequential ASTs to guide the length of time between repeat ASTs [9]. Such thresholds can aid binary choices but cannot estimate the likelihood of passing an AST to individual patients. The calculation of baseline early-morning cortisol to ACTH ratio (cortisol/ACTH) has been proposed as a screening test and been shown to effectively discriminate primary AI, but is unable to distinguish between secondary AI and normal adrenal function [19].

We set out to use real-world data from sequential ASTs to investigate whether the results of a previous test could be combined with a new early-morning cortisol and cortisol/ACTH ratio to predict the result of a new AST. We validate the model in prospective data from the same center not available at the time of model development. This novel approach has the potential to improve individual patient decisions and lead to cost savings by reducing the numbers of ASTs performed.

## Materials and Methods

### Study Design and Participants

To develop the prediction model, we retrospectively reviewed all AST results between August 2016 and September 2021 (the development dataset) at the Endocrine Unit, Sheffield Teaching Hospitals National Health Service (NHS) Foundation Trust, a tertiary adult endocrinology unit in the UK. Following development of the model, we then reviewed all AST results between October 2021 and October 2022 (the validation dataset) at the same center and used these results to validate the model. Patients with results from 2 or more ASTs within the model development period were included for development, regardless of underlying etiology. Patients with the results of a new test conducted within the validation period who also had results of a previous test were included for model validation.

All ASTs were carried out during routine clinical care by specialized endocrine nurses to test for AI. In patients that were taking exogenous glucocorticoids, they were asked to omit glucocorticoids the evening before and morning of the test until completion of the AST. We only assessed patients prescribed oral glucocorticoids if they were on physiological doses of hydrocortisone (≤25 mg/day) or prednisolone (≤5 mg/day). We excluded patients with known protein-losing disorders or severe liver disease, uncontrolled active infections, on estrogens or pregnant, those on strong inhibitors or inducers of p450 CYP3A4, and those who had worked a night shift within the previous week.

### Procedures

Patients attending for an AST had a cannula inserted and baseline serum cortisol and plasma ACTH measured. Appointments were scheduled between 09:00 hours and 09:30 hours, although the precise time of test was variable due to use of real-world healthcare data. We administered 250 µg of Synacthen (Atnahs Pharma UK Limited, Essex, UK) via intravenous injection and measured a serum cortisol 30 minutes after stimulation. We analyzed samples using the Elecsys Cortisol II assay (Cobas, interassay precision coefficient of variation 1.1-5.5%) and Siemens Immulite 2000 (Siemens, Frimley, UK, interassay precision coefficient of variation 6.1-10.0%) for ACTH, and employed a cutoff peak cortisol of ≥430 nmol/L to classify patients as adrenally sufficient and <430 nmol/L as having AI. Endocrinology specialists interpreted the AST result to decide on replacement glucocorticoid requirement, and whether and when to repeat the AST. This was a retrospective longitudinal study of pseudonymized patient data, and thus ethics approval was not required. The study was approved and registered as an institutional case notes review at Sheffield Teaching Hospitals NHS Foundation Trust (registration number 10195).

### Candidate Variables

We used data from sequential ASTs within the same patient to predict the outcome variable of serum cortisol in nmol/L 30 minutes after administration of Synacthen at the more recent test (hereafter “new test”). Candidate predictor variables were the 9 Am measurement of cortisol and ACTH from the new test, number of days between tests, the 9 Am measurement of cortisol and ACTH from the test prior to the new test (hereafter “previous test”), and the 30-minute cortisol from the previous test. The cortisol/ACTH ratio was calculated for both tests by dividing baseline cortisol in nmol/L by baseline ACTH in ng/L [19]. This provided 8 candidate variables to assess to predict the 30-minute cortisol of the new test (Table 1). As measurement of plasma ACTH at baseline when performing the AST is not universal practice, we repeated the process using the 5 predictor variables that did not include plasma ACTH.

**Table 1.** Descriptive statistics and missing values of all candidate variables

	Development dataset		Validation dataset	
Parameter	Missing values (n)	Mean (SD) (Range)	Missing values (n)	Mean (SD) (Range)
Previous baseline cortisol (nmol/L)	0	186.0 (109.9) (2 to 664)	0	148.6 (112.7) (3 to 471)
Previous baseline ACTH (ng/L)	21	27.6 (22.7) (2 to 213)	0	29.9 (37.2) (1 to 276)
Previous baseline cortisol/ACTH ratio (nmol/ng)	21	9.1 (6.5) (0.3 to 38.8)	0	7.7 (9.0) (0.3 to 79.0)
Previous cortisol 30 minutes after Synacthen (nmol/L)	0	360.4 (177.2) (12 to 826)	0	311.0 (195.9) (25 to 808)
Time between tests (days)	0	318 (249) (18 to 1379)	0	483 (280) (86 to 1582)
New baseline cortisol (nmol/L)	0	205.6 (131.3) (2 to 836)	0	170.4 (121.1) (3 to 516)
New baseline ACTH (ng/L)	22	28.5 (27.5) (1 to 276)	0	25.7 (29.7) (3 to 232)
New baseline cortisol/ACTH ratio (nmol/ng)	22	10.0 (9.6) (0.3 to 83.6)	0	11.9 (16.6) (0.5 to 96.3)
Dependent variable				
New cortisol 30 minutes after Synacthen (nmol/L)	0	387.6 (202.0) (9 to 1157)	0	343.5 (207.9) (22 to 1134)
Time of day test conducted: (not tested for parameter inclusion in modeling)				
Time of day: baseline measurements of new test	0	9:57 Am (55 minutes) (8:15 to 13:50)	0	10:06 (71 minutes) (8:15 to 14:05)
Time of day: baseline measurements of previous test	0	9:52 Am (48 minutes) (8:18 to 13:50)	0	10:07 (53 minutes) (8:15 to 12:45)

Abbreviations: ACTH, adrenocorticotropin hormone.

### Model Comparisons

**Figure 1.**
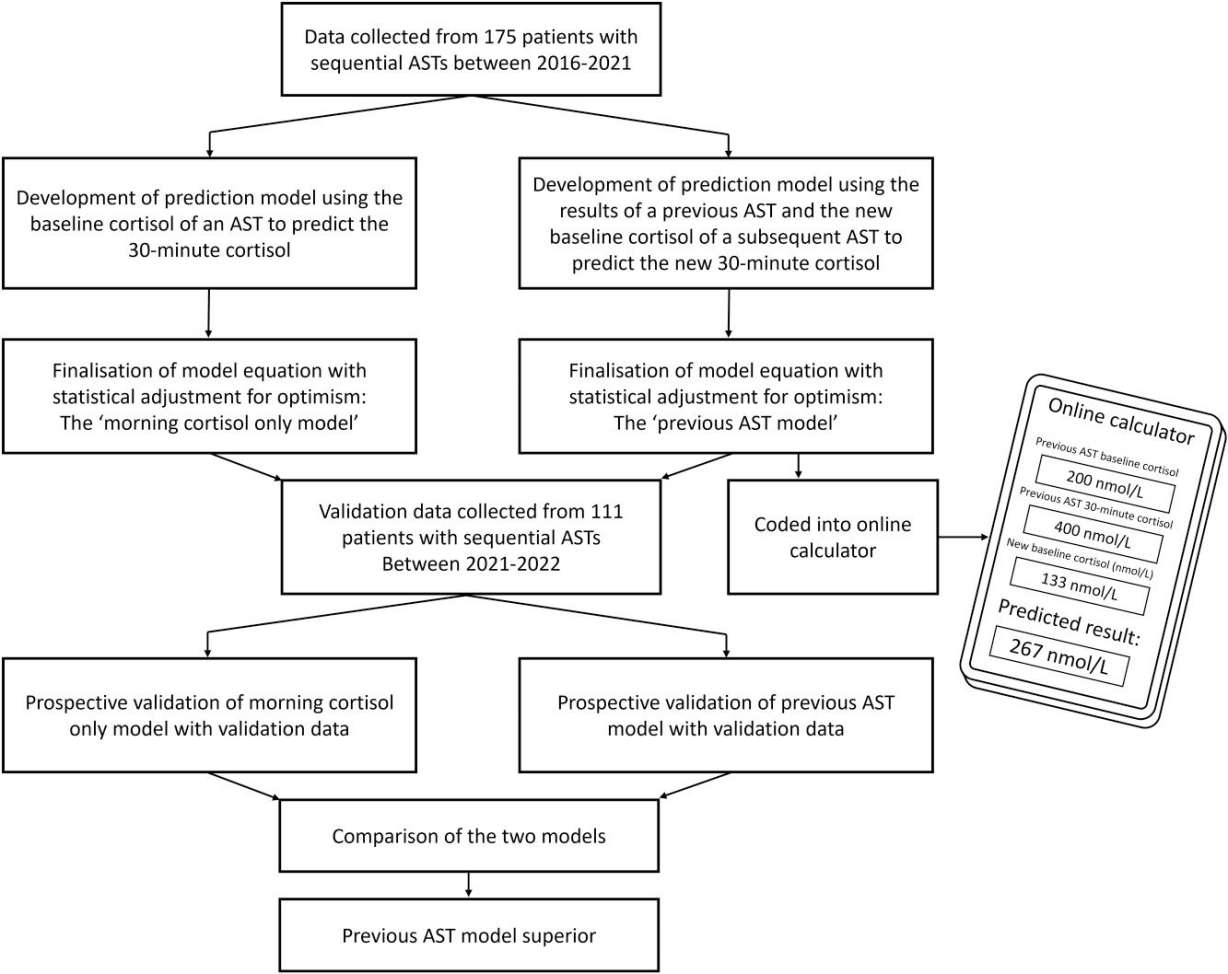
Study flow diagram. AST, adrenocorticotropin hormone stimulation test.

To quantify the improvement to post-Synacthen 30-minute cortisol predictions provided by our model (hereafter “previous AST model”), we compared this with a model, developed following the same methodology, using only new morning baseline cortisol (without previous Synacthen test results) to predict new post-Synacthen 30-minute cortisol (hereafter “morning cortisol only model”). Models were compared by the likelihood ratio test and incremental proportion of variance explained by predictions (ie, improvement in *R*^2^) (Fig. 1).

### Statistical Methods

We analyzed data using R: A language and environment for statistical computing, packages detailed in supplementary material (Table S1 [20]) (R Core Team, R Foundation for Statistical Computing, Vienna, Austria, https://www.R-project.org/). Prior to model development we carried out a power analysis as defined by the methodology of *Riley et al* to ensure sufficient data for reliable estimation of a prediction model [21]. This study is reported according to the transparent reporting standards recommended by the equator network for prediction models (see TRIPOD checklist [20, 22]).

To produce the prediction model, we used bootstrapping with replacement to create 5000 versions of the development dataset. We imputed missing data by random forest within each replicated dataset [23, 24]. We used multiple regression with backwards selection of our 8 candidate variables: previous test baseline ACTH, previous test baseline cortisol, previous test 30-minute cortisol, days between tests, new baseline ACTH, new baseline cortisol, and both calculated cortisol/ACTH ratios. We assessed nonlinear relationships with fractional polynomial transformations. We adjusted regression coefficients estimated by the regression analysis for optimism by parameter-wise shrinkage. To identify the most predictive variables, we repeated this process in each bootstrapped version of the data, removing variables with a regression coefficient *P* > .05. We retained a candidate variable in the final aggregated model if it was statistically significant in over 90% of the replicated datasets (bootstrap inclusion fraction >0.9) [25-27].

We created the final model formulae by taking the mean of the optimism-adjusted coefficients from each of the bootstrap replications. We applied the final model on each bootstrap replicated dataset to create internal validation performance statistics by assessing the calibration of the model (see statistics methods [20]). We used receiver operator characteristics (ROC) analysis to assess discrimination at the threshold of 430 nmol/L for a pass by calculating area under the curve (AUC).

### Cross Validation Method

We used cross validation to assess the impact of the heterogeneity of the patient population on model specification using similar regression analysis with bootstrapping and parameter-wise shrinkage. We developed the model excluding 1 group defined sequentially by sex, diagnosis, or steroid treatment, and tested the derived model on the excluded group at each iteration [28]. We used data from patients who had more than 2 ASTs to assess the impact of heterogeneity of time between tests on model validity, developing the models using results from the most recent AST and the AST directly before, and comparing predictions calculated using the most recent AST and results of ASTs carried out 2 or 3 tests previously, and vice versa.

### Sheffield Short Synacthen Calculator

We coded the final model formula into an online calculator using the “*Shiny*” R package, designed to allow external validation of the model. The calculator also links to a spreadsheet that allows the entry of multiple patient values, and that can allow creation of external fit statistics. This allows assessment by external researchers prior to being suitable for clinical utilization.

### Prospective Validation

Parameters of the prediction model were fixed and shared with all coinvestigators in June 2022. The validation dataset was collected after finalization of the prediction model, comprising new tests conducted between October 2021 and October 2022, after which local policy for repeat ASTs changed due to the employment of salivary cortisone testing to exclude AI [29]. The model development was thus done completely blinded to the dataset used for prospective validation. To assess the clinical benefit of employing the prediction model, we calculated the proportion of ASTs that would have been saved in the validation dataset at different thresholds of predicted values that would equate to varying levels of sensitivity and specificity.

## Results

### Study Population

The development dataset contained information from 258 sequential ASTs, performed on 175 different patients (55.4% female) aged between 18 and 84 years (mean 52.4 years, SD 16.4), with complete data on 218 sequential tests (Table 1). Patient numbers by sex, diagnosis, and current treatment are in Table 2. Results of the power analysis against all 4 recommended criteria [21] showed adequate data for model development (Supplementary Calculations 1 [20]).

**Table 2.** Demographics of total cohort and subgroups within the development and validation datasets

Subgroups	Development dataset		Validation dataset	
	Number of patients	Age (years): Mean (SD) (Range)	Number of patients	Age (years): Mean (SD) (Range)
Total	175	52.4 (16.4) (18 to 84)	111	51.8 (17.5) (17 to 88)
Sex of patients				
Male	78	55.8 (16.7) (18 to 84)	45	53.4 (17.0) (18 to 82)
Female	97	49.7 (15.8) (19 to 83)	66	50.6 (17.8) (17 to 88)
Type of AI				
Adrenal sufficient	8	39.3 (19.5) (19 to 65)	32	45.9 (16.6) (18 to 88)
Primary AI	5	41.6 (18.0) (18 to 64)	1	37
Secondary AI	53	50.7 (16.6) (20 to 82)	12	46.3 (16.8) (17 to 74)
Tertiary AI	109	54.7 (15.5) (18 to 84)	65	56.2 (17.1) (18 to 86)
Missing type of AI	—	—	1	31
Delivery of glucocorticoid replacement				
Oral	61	53.9 (15.7) (18 to 83)	47	48.2 (17.7) (17 to 83)
Inhaler	16	54.7 (15.4) (28 to 84)	1	46
Both oral and inhaled	47	55.9 (15.1) (22 to 82)	35	62.1 (12.1) (37 to 86)
None	51	46.8 (17.8) (19 to 82)	27	46.0 (17.3) (18 to 88)
Intra-articular	0	—	1	18
Formulation of oral glucocorticoid replacement				
Hydrocortisone	56	53.1 (15.8) (18 to 82)	34	45.2 (18.7) (17 to 83)
Prednisolone	52	57.3 (15.0) (20 to 84)	11	53.8 (11.6) (37 to 76)
Both hydrocortisone and prednisolone	0	—	2	68.5 (5.0) (65 to 72)

Abbreviations: AI, adrenal insufficiency.

### “Morning Cortisol Only Model” Development

To develop the “morning cortisol only model,” all 516 ASTs were used across 5000 bootstrap replications of the development dataset and resulted in the same optimum fractional polynomial transformation of the square root of the new baseline cortisol divided by 100. Following parameter-wise shrinkage and bootstrap aggregation, the final morning cortisol only model formula was:


$$Morning\;cortisol\;only\;model\;:\;\mathrm{New}\;30\text{-}\mathrm{minute}\;\mathrm{cortisol}\;=\;-54.5+\;325.1\sqrt{\left(\frac{\mathrm{New}\;\mathrm{baseline}\;\mathrm{cortisol}}{100}\right)}$$

**Figure 2.**
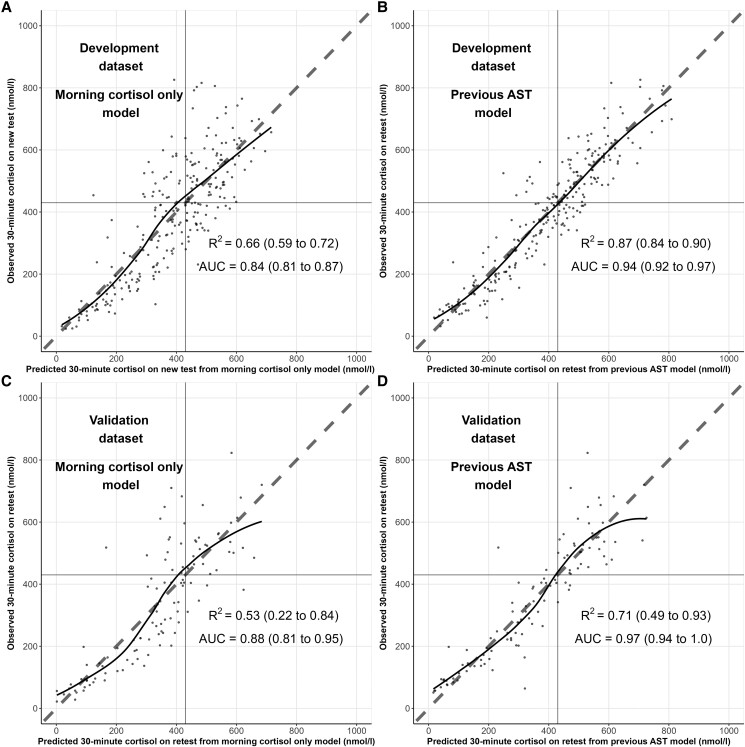
Calibration of prediction models. Calibration plots of morning cortisol only model (A, C) and previous AST model (B, D) on development data (A, B) and validation data (C, D). Perpendicular solid lines drawn to indicate the pass threshold of AST (430 nmol/L). Dashed 45° line indicates the line of identity (x = y). Points are the observed values in the original dataset plotted against predicted values. Curved line through points is locally estimated scatterplot smoothing curve. AST, adrenocorticotropin hormone stimulation test; AUC, area under the receiver operated characteristic curve.

**Figure 3.**
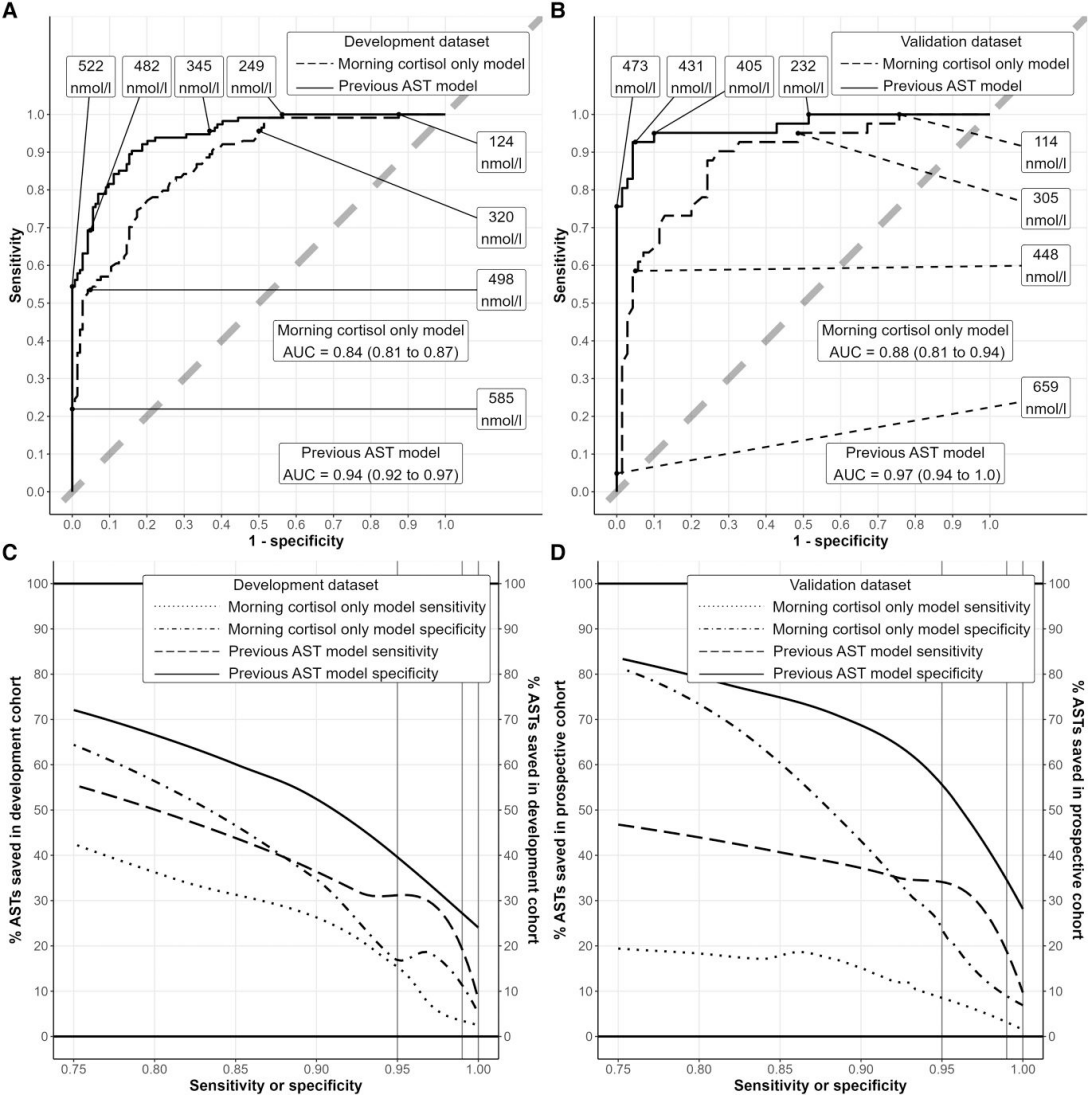
Discrimination and clinical benefit of employing prediction models. (A) Receiver operated characteristic (ROC) plot for morning cortisol only model and previous AST model on development dataset. (B) ROC plot for morning cortisol only model and previous AST model on validation dataset. Points are labeled on ROC plots with the predicted value of 30-minute serum cortisol from models that 1 would need to apply to attain the corresponding sensitivity and specificity read from the axes. Plot B shows that assuming a pass in anyone predicted over 473 nmol/L from the previous AST model would maintain a specificity of 100% in the validation dataset, with a sensitivity of 75.6%, compared with assuming a pass in anyone predicted over 659 nmol/L from the morning cortisol only model to maintain specificity of 100% and achieving a corresponding sensitivity of 4.9%. (C) Benefit plot from development dataset, showing trade-off between sensitivity and specificity and proportion of tests saved via locally estimated scatterplot smoothing curves, if employing a policy where only patients within an equivocal range of predictions proceed to AST. Combine the proportion of tests saved read from the y axis for each of permitted sensitivity and specificity set by the user to obtain overall proportion of tests saved. (D) Benefit plot from validation dataset. ROC, receiver operated characteristics. AST, adrenocorticotropin hormone stimulation test.

Performance statistics for the morning cortisol only model (Table 3) show an internal *R*^2^ of 0.66 (95% CI 0.59-0.72, Fig. 2) and AUC of 0.84 (0.81-0.87, Fig. 3). The baseline cortisol that would predict a 30-minute cortisol of exactly the pass threshold of 430 nmol/L equates to 133 nmol/L. The morning cortisol only model therefore estimates a patient with a new baseline cortisol of 133 nmol/L as having a 50% likelihood of passing the new AST.

**Table 3.** Multiple regression model coefficients and performance statistics for morning cortisol only model

Parameter	Parameter-wise shrunken estimate	Standard error of shrunken estimate	Shrinkage factor	*P* value of parameter
Intercept	−54.476	13.510	0.9890	0.0073
β_1_	325.134	10.480	0.9987	1.808 ×10^−90^
**Performance measure**	**Development dataset**		**Validation dataset**	
*R* ^2^	0.656 (0.593-0.718)		0.526 (0.218-0.834)	
Root mean squared error	110.3 (99.1-121.5)		140.9 (78.7-203.0)	
Calibration slope	1.00 (0.94-1.06)		0.92 (0.73-1.09)	
Calibration Intercept	−0.5 (23.9-22.9)		39.0 (−36.8 to 114.7)	
AUC at pass threshold of 430 nmol/L	0.839 (0.806-0.873)		0.879 (0.812-0.945)	

Numbers in brackets correspond to 95% CI. Parameters correspond to morning cortisol only model: 30-minute cortisol = Intercept + β_1_ × √(baseline cortisol/100).

Abbreviation: AUC, area under receiver operated characteristics curve.

### “Previous AST Model” Development

In our development of the “previous AST model,” days between tests did not improve the accuracy of predictions, having a regression coefficient with *P* < .05 in only 69% of bootstrap replications, and were thus removed (Table S2 [20]). The most appropriate candidate variable transformations produced the following previous AST model formula, which includes previous AST results and new baseline cortisol (Table S3 [20]):


$$Previous\;AST\;model:\;\mathrm{New}\;30\text{-}\mathrm{minute}\;\mathrm{cortisol}\;=\;-116.5+\;251.5\sqrt{\left(\frac{\mathrm{New}\;\mathrm{baseline}\;\mathrm{cortisol}}{100}\right)}-49.4\left(\frac{\mathrm{Previous}\;\mathrm{baseline}\;\mathrm{cortisol}}{100}\right)+\;714.3\left(\frac{\mathrm{Previous}\;30\text{-}\mathrm{minute}\;\mathrm{cortisol}}{1000}\right)$$

Performance statistics for the previous AST model (Table 4) show an internal *R*^2^ of 0.87 (0.84-0.90, Fig. 2) and AUC of 0.94 (0.92-0.97, Fig. 3). New baseline cortisol predictions are adjusted by the results of a previous test using the previous AST model. In the context of a previous baseline cortisol of 100 nmol/L and previous 30-minute cortisol of 200 nmol/L, a new baseline cortisol of 133 nmol/L predicts a new 30-minute cortisol of 267 nmol/L. In the context of a previous baseline cortisol of 200 nmol/L and previous 30-minute cortisol of 400 nmol/L, a new baseline cortisol of 133 nmol/L predicts a new 30-minute cortisol of 360 nmol/L.

**Table 4.** Multiple regression model coefficients and performance statistics for previous AST model

Parameter	Parameter-wise shrunken estimate	Standard error of shrunken estimate	Shrinkage factor	*P* value of parameter
Intercept	−116.5	14.87	0.994	1.27 × 10^−09^
β_1_	251.5	15.34	0.997	2.86 × 10^−44^
β_2_	−49.4	7.11	0.976	6.97 × 10^−07^
β_3_	714.3	48.51	0.993	1.88 × 10^−25^
**Performance measure**	**Development dataset**		**Validation dataset**	
*R* ^2^	0.866 (0.836-0.896)		0.711 (0.492-0.930)	
Root mean squared error	73.4 (63.5-83.2)		109.6 (54.3-164.8)	
Calibration slope	1.00 (0.95-1.06)		0.92 (0.84-0.99)	
Calibration intercept	−0.9 (−21.1 to 19.2)		37.5 (7.0-68.1)	
AUC at pass threshold of 430 nmol/L	0.944 (0.919-0.969)		0.969 (0.937-0.998)	

Numbers in brackets correspond to 95% CI. Parameters correspond to previous AST model: 30-minute cortisol = Intercept + β_1_ × √(new baseline cortisol/100) + β_2_×(previous baseline cortisol/100) + β_3_×(previous 30-minute cortisol/1000).

Abbreviation: AUC, area under receiver operated characteristics curve.

We report comprehensive assessment of the inclusion of ACTH in the supplementary material (Fig. S1 [20]), as ACTH did not make a clinically significant improvement to predictions.

### Cross Validation

The original dataset comprised a heterogeneous patient group (Table 2). The calibration slope, calibration intercept, *R*^2^, and AUC for each cross validated version of both the morning cortisol only model and previous AST model are shown in the supplementary material (Table S4 [20]). The best way to interpret the cross validation is by visual inspection of the calibration plots (Fig. S2 [20]). These show the morning cortisol only model is robust between sexes, but not robust between type of glucocorticoid replacement or diagnostic category (poorest performance statistics when validated on those not taking steroids: *R*^2^ = −0.58, AUC = 0.79). In comparison, the calibration fit of the previous AST model is consistently closer to the optimum prediction line of x = y showing the previous AST model is robust when cross validated in both sexes, different steroid formulations, different etiologies or whether another AST had been conducted in between the 2 ASTs used for prediction (poorest performance statistics: *R*^2^ = 0.72, AUC = 0.92).

The model developed from test results from the most recent test performed prior to the predicted test (n = 258, mean 347 days between tests, SD 263) remained effective when tested using parameters from tests 2 or 3 tests prior to the predicted result (n = 160, mean 675 days between tests, SD 346) (*R*^2^ = 0.86, AUC = 0.92).

### Prospective Validation

The patient cohort in the validation dataset included 111 sequential tests. Of these, 67 patients were different patients from those who had contributed data to the development of the model. The other 44 patients had contributed previous sequential test data to model development and had since undergone another AST providing a new test result that could be used for validation. Mean age was 51.8 years (SD 17.5) (Table 2). Prospective validation of the supplementary ACTH previous AST model was also undertaken and reported in the supplementary material (Table S5 [20]). This patient sample is greater than the minimum 100 recommended for external validation of a prediction model with a continuous outcome by Harrell et al [30].

### Model Comparisons

In prospective validation, morning cortisol only model predictions exhibited calibration of *R*^2^ = 0.53 (0.22-0.84), calibration slope = 0.91 (0.73-1.09), calibration intercept = 40.0 (−36.8 to 114.7), with AUC = 0.88 (0.81-0.95). Previous AST model predictions outperformed these with *R*^2^ = 0.71 (0.49-0.93), calibration slope = 0.92 (0.84-0.99), calibration intercept = 37.5 (7.0-68.1), with AUC = 0.97 (0.94-1.0). The likelihood ratio test confirmed superiority of the previous AST model on validation data (χ^2^ = 80.0, *P* < .001), with marginal improvement in variance explained of 18%.

Employing a policy where one accepts a prediction lower than the threshold for 95% sensitivity as a diagnosis of AI and a prediction higher than the threshold for 95% specificity as adequate adrenal function, with only those in the equivocal range proceeding to an AST would reduce the number of tests performed while retaining acceptable accuracy. This would equate to applying a range of predictions from the morning cortisol only model of 166 nmol/L to 630 nmol/L, saving 23.4% of tests. Applying the previous AST model instead would require an equivocal range of predictions between 430 nmol/L and 470 nmol/L and save 91.9% of tests. Adjusting the threshold sensitivity and specificity employed would alter the proportion of tests saved, as dictated by the benefit curves in Fig. 3, which show sacrificing specificity (increasing proportion of patients incorrectly predicted to pass the AST who were observed to fail) leads to a greater proportion of tests saved than sacrificing sensitivity (increasing proportion of patients incorrectly predicted to fail who were observed to pass).

## Discussion

We have used real-world data from ASTs carried out over 5 years to develop a prediction model that uses cortisol measurements from a previous AST in combination with the baseline cortisol of a new AST to predict the result of cortisol measured 30 minutes after Synacthen on the new AST. When compared with a model that employs only the new baseline cortisol to predict the result of the new AST, the test incorporating previous test measurements is superior, and can provide bespoke predictions. If results of a previous AST are available, they can be objectively combined with the results of any new early-morning cortisol using a multivariable prediction model to improve the accuracy of the predicted AST result, to inform the likelihood of an individual patient passing a repeat AST.

To date, focus has been on defining threshold values for early-morning cortisol to reduce numbers of ASTs. Different thresholds have variable predictive accuracy, often determined by the discrimination provided by the cohort from which they were developed, rather than being validated in external patient cohorts. Our cross validation showed that using the new baseline cortisol alone to predict the result of a new AST does provide relatively accurate predictions but is inconsistent between those with different causes of AI and on different steroid formulations. However, employing the previous test cortisol measurements alongside the new baseline cortisol using multiple regression improves the consistency of predictions across different indications for the test. The previous AST results are thus acting as proxy variables that provide insight into the underlying relationship of the individual patients’ hypothalamo–pituitary–adrenal axis and its likely response to Synacthen. Allowing the original continuous variables themselves to inform the prediction model, rather than attempting to develop strict and sometimes contentious categories for patients, is extracting the most value from the dataset and leads to highly accurate personalized predictions. The previous AST model presented here informs upon the likelihood of passing, with higher predictions consistent with a higher likelihood of passing, which cannot be provided when employing a binary threshold.

While ACTH incorporated into the prediction model as the cortisol/ACTH ratio did show statistical significance, the overall predictive accuracy of the supplementary model that incorporated ACTH only marginally improved predictions in the development dataset (*R*^2^ = 0.88 vs 0.87) and in the validation dataset (*R*^2^ = 0.72 vs 0.71) [20]. While ACTH is valuable for differentiating primary from secondary AI, such marginal improvement in predictions does not warrant the additional effort and cost in measuring the variable in patients who are being considered for a repeat AST. Nonetheless, the statistical significance in our modeling warrants further investigation of the cortisol/ACTH ratio in other studies investigating the monitoring or recovery of patients with AI.

Days between ASTs did not improve the prediction of the result of the new AST. This was further emphasized by cross validation where the previous AST model was robust when tested using historical test results averaging 2 years prior to the new test compared with predictions developed using historical test results averaging 1 year prior to the new test. This is consistent with adrenal function recovering at different rates between patients or failing to recover at all. We have therefore not provided empirical evidence about how long to wait prior to measuring a new early-morning cortisol in patients before deciding to conduct another AST. Instead, this model offers an objective method to increase the predictive power of a new early-morning cortisol in patients who have undergone a previous AST, a practice currently employed but reliant upon a clinician's subjective judgement. This method has the potential to be introduced into clinical practice by taking the Sheffield Short Synacthen Calculator and validating it using sequential test results from at least 100 patients within a single center [30]. When validating this model, one must be careful not to apply it to patients who had their previous AST measured on a different assay from the recent AST measurement, as the relationship between baseline cortisol and 30-minute cortisol on different assays will be different, with cortisol measurements from different assays having been shown to be up to 39% different in magnitude [17]. Once validated, the accuracy of the model on local data can be used to calculate appropriate prediction intervals, which can in turn be used to convert prediction results into the probability of passing a repeat AST in the context of the local pass threshold. These predictions and probabilities could be discussed with patients to facilitate patient-centered decision-making as to the timing of a repeat AST. Alternatively, local guidelines could incorporate these improved predictions to facilitate specific prediction thresholds where patients could either be declared adrenally insufficient, adrenal sufficient, or equivocal and in need of an AST prior to any change of management.

The limitations of this study include model development using results from a single center, where only cortisol 30 minutes after administration of Synacthen is measured. While the validation data have allowed external validation according to the definition that the data were not available at the time of model development, it has not shown the model to work in a different center, or in another assay. Some other centers measure cortisol 60 minutes after Synacthen; use of a 60-minute value would require further validation. We were unable to assess dose duration in those receiving glucocorticoid therapy within modeling, or account for other biochemical variables that were not measured at our center that may be predictive of the result of an AST such as albumin. There were missing data, but this has been appropriately accounted for with random forest imputation as recommended to reduce the risk of bias in model development [22, 23, 31]. We would encourage work by a separate research team to validate these formulae in other centers using different assays.

In our center, we now employ early-morning salivary cortisone to exclude AI, with only values in the equivocal range of 7 to 17 nmol/L proceeding to a repeat AST [29]. As we have shown with early-morning serum cortisol in this study, we hypothesize that combining the result of an early-morning salivary cortisone with previous AST results, if available, may improve the predictive accuracy of salivary cortisone and will be investigated in future work. Employing noninvasive testing alongside transparent mathematical algorithms employed through easy to use web applications has enormous potential to generate further cost savings by the reduction of the increasing burden of dynamic endocrine testing on healthcare systems, in particular in resource limited low- and middle-income countries [32].

Our study has demonstrated that the result of an AST can be predicted using the results of previous ASTs performed up to 5 years earlier alongside a new early morning cortisol. This indicates future work employing longitudinal repeated measures modeling of cortisol values within the same patients may allow for more detailed and precise predictions of the time or likelihood of future adrenal recovery. Similarly, repeated measures of noninvasive markers such as salivary cortisone could increase their value in clinical practice even further and should be investigated in future research to improve repeat testing protocols and inform glucocorticoid weaning regimes.

### Conclusion

Results of a previous AST can be objectively combined with a new early-morning cortisol to predict the results of a new AST with greater accuracy than the new early-morning cortisol alone. This model can be validated using data from different assays in other centers using the Sheffield Short Synacthen Calculator available at endocrinology.shinyapps.io/sheffield_sst_calculator/. Once validated, this approach has the potential to offer greater objectivity in the assessment of new early-morning cortisol to diagnose adrenal insufficiency or help inform the timing of future ASTs to reduce the burden of repeat dynamic testing on the healthcare system.

## Data Availability

Data were collected for this study in anonymized form as part of service evaluation, and is therefore not available on a public repository, and will not be shared on an individual patient level. Local service evaluation should be carried using the Sheffield Short Synacthen Calculator prior to use for clinical decisions. If predicted values are shown to be miscalibrated, we recommend the use of model updating methods before use in clinical practice [33]. Please contact the corresponding author if you would like support with this process.

## Abbreviations

ACTH: adrenocorticotropin hormone
AST: adrenocorticotropin stimulation test
AI: adrenal insufficiency
AUC: area under the curve
ROC: receiver operator characteristics
